# British Medicinal Plants*By an oversight, this article was omitted from its proper place in the series of papers on British Medicinal Plants.—Ed.

**Published:** 1892-04

**Authors:** Alfred Heath

**Affiliations:** London, England


### BRITISH MEDICINAL PLANTS.*

ALFRED HEATH, M. D., F. L. S., LONDON, ENGLAND.

### ORDER 26. ROSACEÆ (CONTINUED).

*Fragraria vesica* (from Fragro, to smell sweet). The Wood Strawberry.—The mature fruit of this plant was formerly recommended in gouty and calculous affections, probably on account of its efficacy in removing tartar from the teeth, which it does very effectually. To ladies, and those who wish for good and clean teeth, there is nothing better than cleaning them with ripe strawberries, and it is a *perfectly safe* dentifrice. As every one knows, it is a most delicious fruit, and, if sound, one can scarcely eat too many. It has been called the "oyster of summer." The leaves of strawberry, boiled and applied to wounds, are said to take away the burning heat; a decoction from the plant strengthens the gums, and fastens loose teeth, and is said to be good against inflammations of the mouth and gums; it is reputed to be effectual in regulating or staying bloody fluxes or other issues of blood. The fruit quenches thirst and allays inflammation or heat of the stomach. The distilled water is said to be good against passion of the heart (sadness), reviving the spirits, and making the heart light. It is said to cleanse the

* By an oversight, this article was omitted from its proper place in the series of papers on British Medicinal Plants.—ED.

face from redness and spots, and to make the skin fair and smooth. It has been used with success against stone in the kidneys. In the United States, strawberries may be obtained from January to December. In the winter months they come from the Southern States, in the summer from the Northern States, so that American ladies can always have this useful and delicious fruit, with its many virtues. There is some proving in Allen's *Materia Medica.*

*Geum urbanum* (Wood Avens).—The root of avens has a fragrant, spicy smell ; a small quantity is sometimes put into ale to give it a fine flavor. The root has been used to tan leather ; also, to dye wool, to which it gives a permanent yellow color. The avens is not much used in medicine, but the powdered root is said to be equal to Peruvian bark in ague. It is also recommended in dysentery and disorders arising from a weak and relaxed state of the bowels. I believe there is no proving.

Orders 27 and 28 contain no British plants used in Homœopathy.

### Order 29.—Onagraceæ.

*Epilobium palustre* (Willow Herb).—This plant is said to be cooling and drying, and good to stay all kinds of fluxes and looseness ; it is said to be a remedy in venereal discharges, nocturnal pollutions, etc. The leaves are cooling when applied to hot tumors and inflammations. There is some proving in Dr. Allen's *Materia Medica.*

*Œnothera biennis* (Evening Primrose).—A naturalized American plant, very common, especially in gardens ; brought to this country from Virginia in 1680. The fleshy roots were formerly cultivated as a vegetable ; eaten raw, they were said to increase the relish and desire for wine, hence, its generic name, meaning *oinos*, wine, and *thera*, a hunt or pursuit. This plant does not appear to have been used in medicine until its introduction by Homœopathy. There is some proving in Allen's *Materia Medica.*

Order 30, Haloragaceæ, contains no plants at present used in Homœopathy.

### ORDER 31.—CUCURBITACEÆ.

*Bryonia dioica* (the Red-berried Bryony).—This plant is the only member of the order *cucurbitaceæ* indigenous to Great Britain. It must not be confounded with the well-known *Bryonia alba*, the berries of which are *black*. Both plants contain *Bryonin*, and may have very *similar* medicinal properties, but that is not sufficient reason why one should be substituted for the other, especially with such distinct differences, both in the flowers and fruit. It is surprising that the *British Homœopathic Pharmacopœia* allows either plant to be used. I suppose because it is more easily obtained, in spite of the fact that *Bryonia alba* was the one proved. All other pharmacopœias draw attention to the necessity of using the black-berried plant. The *American Homœopathic Pharmacopœia* (Tafel) draws especial notice to the necessity of using the plant proved, but unfortunately most if not all the tincture of Bryonia used is made in Germany, and as the red-berried Bryony grows there also, there is no question that it is often used either separately or with the black-berried plant in making the tincture. I would advise every homœopathic chemist to write to Germany or France for the *black berries* and grow the plants, but unless they obtain the entire berry there is no certainty that the *seeds*, which are supplied separated from the pulp and skin, will turn out true. My first trials in this direction resulted in failure, although obtained from the most eminent seed-houses, after *two years'* growth they berried *red*. I have been obliged to go thus fully into this matter under *Bryonia dioica* because the *Bryonia alba* is not an English plant, and could not well be brought into this paper; it ought to be English, because it grows here when planted, *perfectly*, and because it grows on the European continent from the north to the south; and, further, because it is a homœopathic remedy of such extreme importance, and for this reason the utmost care should be exercised in preparing the tincture. Probably many failures result from not using the genuine plant, and these failures may mean loss of the patient's life. The *Bryonia*

*dioica* produces vomiting and purging, even in small doses. It has been used empirically for the cure of dropsy and hysterical complaints ; poultices of the leaves have been recommended as an excellent remedy applied to painful tumors and *severe rheumatism*, old sores, scrofulous swellings, etc. ; it has been successfully used as a hydragogue, emenagogue, and diuretic, and when fresh it is emetic; it is a powerful and irritating cathartic , it was at one time employed in asthma, mania, and epilepsy ; in large doses it produces inflammation of the bowels and all the other effects of an acrid vegetable poison. These effects are caused chiefly by Bryonin, the active principle. *Bryonia alba* also contains the same substance, and I will mention here some of the effects *produced* by the latter when proved, which will be seen to be the same as those mentioned above, as cured by the drug :

Bryonia produces inflammation of mucous and serous membranes and dropsical conditions, and inflammation of the *lungs*, *pleura*, *heart*, *uterus*, *liver*, *bowels*, *bladder*, etc. ; it is an every-day remedy in the treatment of these conditions, as every homœopath and many allopaths know. Martindale, one of the examiners of the Pharmaceutical Society, includes it in his extra *Pharmacopœia* as a remedy for *pleurisy*, *cough*, *dropsy*, etc., given in *small doses*. It also *produces* violent muscular pains like rheumatism, aggravated by the slightest movement, gouty-like pains. It suppresses the menstrual flow, diminishes the secretion of urine, as well as producing the opposite conditions; it produces the most obstinate constipation, as well as the most severe diarrhœa. Its action on the respiratory organs may be imagined, if it is capable of inflaming those organs, yet its action in small doses is wonderful when these conditions arise from natural causes. It produces the most terrible shortness of breath, aggravated by the least movement ; this aggravation from movement is peculiar to Bryonia. It produces terrible delirium, great mental irritability, and excitement, patient desires to get out of bed and go home, desires things that cannot be had, and refuses when offered things that were desired, irrational talking, etc., rush of blood to the head. For the other effects of Bryonia, see the provings in Hering's *Guiding Symptoms.*

ORDER 32.—PORTULACEÆ.

This order contains no British plant used in homœopathic medicine.

